# Flatworms have lost the *ri*ght *o*pen reading frame kinase 3 gene during evolution

**DOI:** 10.1038/srep09417

**Published:** 2015-05-15

**Authors:** Bert Breugelmans, Brendan R. E. Ansell, Neil D. Young, Parisa Amani, Andreas J. Stroehlein, Paul W. Sternberg, Aaron R. Jex, Peter R. Boag, Andreas Hofmann, Robin B. Gasser

**Affiliations:** 1 Faculty of Veterinary and Agricultural Sciences, The University of Melbourne, Parkville, Victoria, Australia; 2 HHMI, Division of Biology, California Institute of Technology, Pasadena, California, USA; 3 Faculty of Medicine, Nursing and Health Sciences, Monash University, Clayton, Victoria, Australia; 4 Structural Chemistry Program, Eskitis Institute, Griffith University, Brisbane, Australia

## Abstract

All multicellular organisms studied to date have three *ri*ght *o*pen reading frame kinase genes (designated *riok-1*, *riok-2* and *riok-3*). Current evidence indicates that *riok-1* and *riok-2* have essential roles in ribosome biosynthesis, and that the *riok-3* gene assists this process. In the present study, we conducted a detailed bioinformatic analysis of the *riok* gene family in 25 parasitic flatworms (platyhelminths) for which extensive genomic and transcriptomic data sets are available. We found that none of the flatworms studied have a *riok-3* gene, which is unprecedented for multicellular organisms. We propose that, unlike in other eukaryotes, the loss of RIOK-3 from flatworms does not result in an evolutionary disadvantage due to the unique biology and physiology of this phylum. We show that the loss of RIOK-3 coincides with a loss of particular proteins associated with essential cellular pathways linked to cell growth and apoptosis. These findings indicate multiple, key regulatory functions of RIOK-3 in other metazoan species. Taking advantage of a known partial crystal structure of human RIOK-1, molecular modelling revealed variability in nucleotide binding sites between flatworm and human RIOK proteins.

All multicellular organisms studied to date have three *ri*ght *o*pen reading frame protein kinase genes (designated *riok-1*, *riok-2* and *riok-3*), whereas unicellular organisms have only two (*riok-1* and *riok-2*). Functional studies of *riok* genes in both unicellular and multicellular model organisms have indicated essential roles for *riok-1* and *riok-2* in ribosome biosynthesis, chromosome stability and/or cell cycle progression^1^,^2^,^3^,^4^,^5^,^6^,^7^,^8^,^9^,^10^,^11^. The third gene member, *riok-3*, has not received much attention, being non-essential, but research has shown that RIOK-3 potentially has multiple roles in cells^12^,^13^,^14^. Most research has suggested a role for RIOK-3 in associating with ribosomal proteins of the small ribosomal subunit (SSU) and shown that a lack of RIOK-3 results in a build-up of immature SSUs (18S or pre-40S) in the cytoplasm^10^,^14^. Additionally, RIOK-3 has been demonstrated to inhibit the activation of the nuclear factor κB (NF-κB) transcription factor by interacting with caspase-10, suggesting a role for *riok-3* in the regulation of apoptosis and/or cell survival^12^. Structural information for the RIOK family is limited to the partially solved crystal structure for *Hsap*-RIOK-1 of humans^11^ and three crystal structures for species outside of the animal kingdom, namely *Aful*-RIOK-1 and *Aful*-RIOK-2 of *Archaeoglobus fuldigus* (Archaea) and *Cthe*-RIOK-2 of *Chaetomium thermophilum* (thermophilic fungus)^8^,^9^. In recent studies, we have curated the *riok* gene family for 12 parasitic roundworms (nematodes), predicted functional domains of parasite RIOK proteins using three-dimensional (3D) structural modelling, and prioritized existing kinase inhibitors for repurposing against RIOK-1 of parasitic nematodes^15^,^16^.

Logically extending these studies^15^,^16^, we conducted a detailed investigation of the *riok* gene family in socioeconomically important parasitic flatworms. Some of these flatworms cause neglected diseases of vertebrates. For example, trematodes (flukes) cause diseases such as schistosomiasis, fascioloiasis, clonorchiasis and opisthorchiasis, affecting hundreds of millions of animals and humans^17^,^18^; several of these trematodes are class I carcinogens^19^,^20^ and can predispose to viral infections^21^,^22^. Additionally, cestodes (tapeworms) cause serious diseases, such as echinococcosis, cysticercosis and coenuriasis, in their intermediate hosts (mammals; e.g., livestock animals and humans)^23^,^24^,^25^.

Recent advances in high-throughput sequencing have led to a substantial expansion in the availability of genomic and transcriptomic data for parasitic flatworms (platyhelminths). To date, draft genomes of five trematodes (*Clonorchis sinensis, Opisthorchis viverrini, Schistosoma haematobium, Schistosoma mansoni* and *Schistosoma japonicum*) and four cestodes (*Echinococcus multilocularis*, *Echinococcus granulosus*, *Taenia* *solium* and *Hymenolepis microstoma*) have been published^26^,^27^,^28^,^29^. There has been no detailed exploration of *rio*k genes/RIOK proteins of flatworms. In the present study, using these datasets, we conducted detailed bioinformatic analyses and characterized *riok* genes and predicted RIOK proteins at the functional and structural levels for selected parasitic flatworms, and investigated their evolutionary relationships within the animal kingdom. We showed that none of the 25 flatworms studied has a *riok-3* gene, a finding that is unprecedented for any multicellular organism. Therefore, we investigated whether (*i*) *riok-3* has been lost from flatworms or gained in ecdysozosoans through a gene duplication event, (*ii*) *riok-3* was lost from flatworms entirely as a result of their unique biology and parasitic life style, and (*iii*) the loss of *riok-3* is the result of a loss of specific kinase/ATPase-regulated pathways linked to ribosomal biosynthesis and/or cell growth. In pursuit of specific drug repurposing or design, we took advantage of a known partial crystal structure of human RIOK-1, and employed a modelling approach to generate and compare the nucleotide binding sites of flatworm and human RIOK proteins.

## Results

### Detailed characterization of *riok* genes in flatworms

Based on the characterized *riok-1*, *-2* and *-3* genes in *Caenorhabditis elegans* (WormBase gene identification codes 00019698, 00013688 and 00014012), we identified partial *rioks* in the draft genomes of nine flatworm species, for which published genomic and transcriptomic data were available. Transcripts were inferred based on predicted amino acid sequences, and matching exons in respective genomes were identified in order to curate full-length genes (Table 1). No full-length *riok* genes were identified in *S*. *japonicum*, as the quality of the genomic data was insufficient to characterize their gene structures. However, the partial *Sjap*-RIOK-1 and *Sjap*-RIOK-2 sequences did encode a putative functional kinase domain and were thus retained for subsequent phylogenetic analyses. The *riok-3* gene was not found in any of the flatworm genomes or represented in transcriptomes.

**Figure 1:**
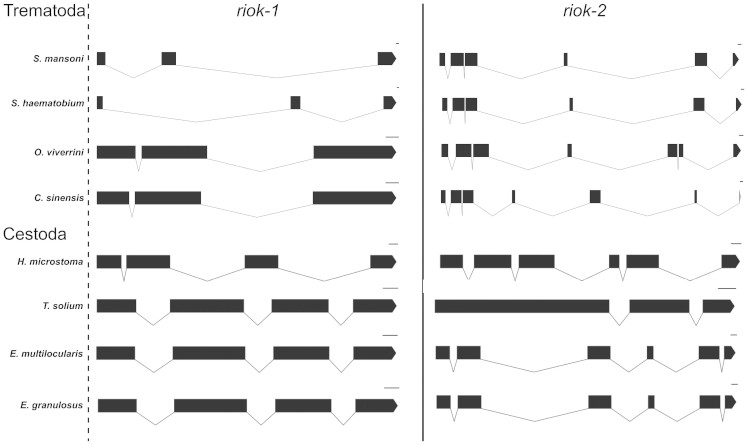
Predicted structures of 16 *riok* genes. Black blocks represent exons and connecting lines represent introns (100 bp scale).

Sixteen full-length *riok* gene structures were defined for the eight flatworms representing trematodes and cestodes (Figure 1). For the trematodes, *riok-1* had an average gene length of 7,422 bp (±3,139 bp; standard error of mean, SEM) and 3 exons. The *riok-2* genes were not significantly longer (8,325 bp ± 646 bp), but had twice as many exons (6–7 exons). For the cestodes, on average, *riok-1* was significantly shorter (2,357 bp ± 345 bp; p < 0.001) than *riok-2* (3,590 bp ± 774 bp). The shorter length of *riok-1* usually coincided with reduced exon numbers. One exception was the *riok-2* gene of *Taenia solium*, which was shorter (1,681 bp) than *Tsol*-*riok-1* (1,989 bp). The mean coding length of all flatworm *riok* genes was 1,444 bp (±16 bp). We did not find a significant relationship between genome size and gene length or exon number.

### Phylogenetic analyses of flatworm RIOKs

**Figure 2:**
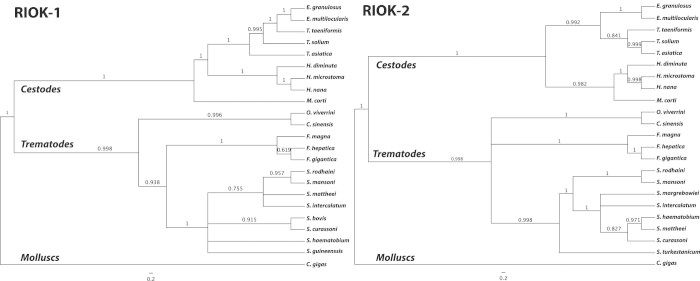
Phylogenetic relationships of RIOK-1 or RIOK-2 amino acid sequences representing 25 species of flatworm studied. Posterior probabilities (pp) are indicated. The scale bar denotes the estimated number of amino acid substitution per site.

Phylogenetic analyses of inferred and curated protein sequence data were used to establish evolutionary relationships for RIOK-1s and RIOK-2s of flatworms (Figure 2; Supplementary Data 1–3). The RIOKs of *Echinococcus granulosus* and *Echinococcus multilocularis* grouped more closely together than to those of *Taenia solium, Taenia taeniaeformis* and *Taenia asiatica*, and all of them grouped together with strong support (pp > 0.99) to the exclusion of RIOKs of *Hymenolepis* *microstoma* and *Mesocestoides corti* (see Figure 2). The RIOKs of *Opisthorchis viverrini* and *Clonorchis sinensis* grouped more closely together than to those of *Fascioloides magna, Fasciola hepatica* and *Fasciola gigantica*, and all of them grouped together with absolute support (pp = 1.0), to the exclusion of RIOKs of *Schistosoma* spp. The two distinct groups of RIOKs of trematodes and cestodes grouped to the exclusion of *Crassotrea gigas* (a molluscan outgroup). Therefore, RIOK-1s and RIOK-2s grouped according to platyhelminth class. There was a positive relationship between exon number and coding length, and evolutionary distance (*P* < 0.001).

### Conservation of the *riok* gene family in metazoans

**Figure 3:**
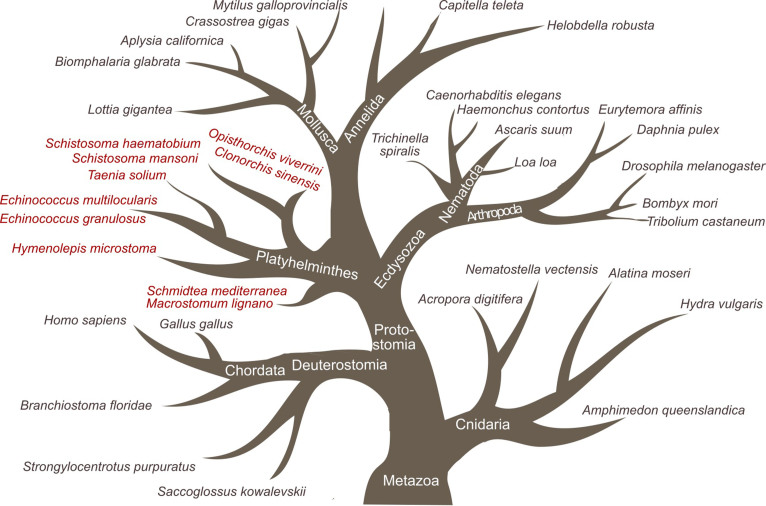
Presence or absence of the *riok-3* gene in/from 45 species representing the Metazoa. Grey indicates species that have three genes (*riok-1* to *riok-3*), and red indicates those that exclusively have genes *riok-1* and *riok-2*.

A bioinformatic analysis of the genomes, transcriptomes and inferred proteomes of 45 species of metazoans (Supplementary Data 4) showed that *riok-3* is present in the earliest metazoans, including cnidarians (*Acropora digitifera* and *Nemostella vectensis*) and poriferans (*Alatina moseri*, *Hydra vulgaris* and *Amphimedon queenslandica*) (Figure 3). The *riok-3* gene was retained in representatives of the deuterostomes including *Saccoglassus kowalevskii, Strongylocentrotus purpuratus, Branchiotoma florida, Gallus gallus* and *Homo sapiens*. The *riok-3* gene is also present in representatives of the three main ecdysozoan lineages including insects (represented by *Drosophila melanogaster, Bombyx mori* and *Tribolium castaneum*), crustaceans (*Euryemora affinis* and *Daphnia pulex*) and nematodes (*Ascaris suum, Caenorhabditis elegans, Haemonchus contortus, Loa loa* and *Trichinella spiralis*). Representatives of two of the three lophotrochozoan lineages including molluscs (*Lottia giganta, Biomphalaria glabrata, Aplysia californica, Crasssostrea gigas* and *Mytillus galloprovincialis*) and annelids (*Capitella telera* and *Helobdella robusta*) have retained *riok-3*, whilst there was no evidence for this gene in either free-living (*Macrostomum ligano, Schmidtea mediterranea, Hymenolepis microstoma*) or parasitic platyhelminths (*Echinococcus granulosus, Taenia solium, Schistosoma haematobium, Schistosoma mansoni, Opisthorchis viverrini* and *Clonorchis sinensis*).

### Conservation of RIOK-3 associating proteins in flatworms

**Figure 4:**
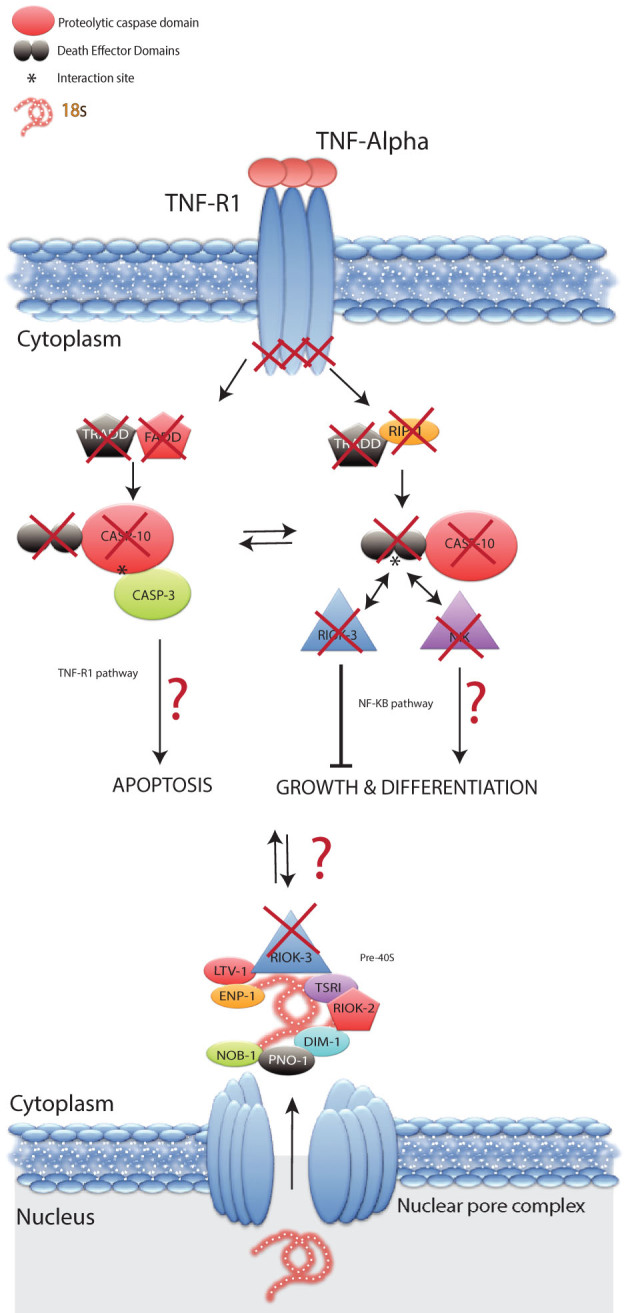
Illustration of flatworm gene conservation, or loss in the tumour necrosis factor pathways and the small subunit ribosomal biosynthesis in flatworms. Red crosses display domains or entire proteins that are present in vertebrates, but have been lost from flatworms, including RIOK-3. The top part of the figure displays gene loss in a simplified representation of the tumour necrosis factor (TNF) pathway. The tumour necrosis factor receptor-1 (TNF-R1) is conserved among flatworms, but lacks death domains (DDs) that interact with the signalling molecules: tumour necrosis factor receptor type 1-associated death domain (TRADD), Fas-associated protein with death domain (FADD) and receptor-interacting protein kinase (RIPK). The downstream caspase-10 enzyme activates apoptosis through a catalytic reaction with other caspases (including caspase-3), and activates growth and differentiation by interacting with NIK (NF-κB-inducing kinase) through its Death Effector Domains (DEDs) via the NF-κB pathway. The bottom part of the figure illustrates the presence of the premature small subunit (SSU, 18S or pre-40S) of nuclear ribosomal RNA and ribosomal proteins as well as the SSU associating proteins (LTV-1, EMP-1, TSR-1, RIOK-2, RIOK-3, DIM-1, PNO-1 and NOB-1) that associate with SSU (pre-40S) in the cytoplasm.

To explain the identified loss of RIOK-3 from flatworms, bioinformatic analyses were performed on the genomes, transcriptomes and inferred proteomes of all available flatworm species, to identify the presence/absence of RIOK-3 associating proteins involved in (a) the biosynthesis of the ribosomal small subunit (SSU), and (b) the tumor necrosis factor receptor-1 (TNF-R1) signalling pathway. Regarding the former, the bioinformatic analysis identified flatworm orthologs for all known metazoan SSU proteins (n = 30) and associating proteins (LTV-1, ENP-1, TSR-1, DIM-1, PNO-1 and NOB-1) essential for the cytoplasmic assembly of SSU (Supplementary Data 5). For the latter pathway, a loss of caspase-10 and the upstream TNF-R1 signalling proteins - Fas-associated protein with death domain (FADD) and T tumor necrosis factor receptor type 1-associated death domain (RADD) protein became evident (Figure 4). Additional gene losses were identified in the NF-κB pathway for the caspase-10-associating receptor-interacting protein kinase (RIPK) and NF-κB-inducing kinase (NIK). In flatworms, we identified TNF-R1 that lacked its cytoplasmic death domains, caspase-3 and caspase-8 (Figure 4).

### Structural analysis and functional predictions of flatworm and host RIOK-1s

**Figure 5:**
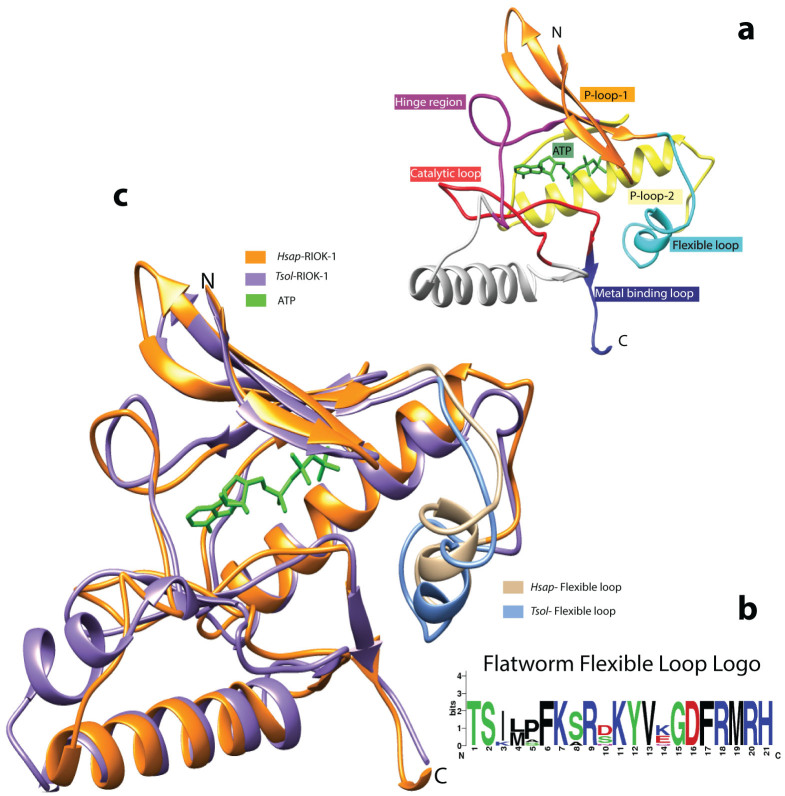
Three-dimensional (3D) structural models for the core kinase region of *Hsap*-RIOK-1 from *Homo sapiens* and *Tsol*-RIOK-1 from *Taenia solium*. Panel a displays the 3D model of *Hsap*-RIOK-1, including the flexible loop. The model is colour-mapped according to the distinct domains and includes the natural ligand ATP (green). Panel b is a logo-representation of the consensus sequence for the flexible loop region of RIOK-1 representing eight different flatworms. Panel c shows superimposed models of *Hsap*-RIOK-1 (orange) and *Tsol*-RIOK-1 (purple), including their natural ligand ATP (green). The flexible loop is colour-mapped for both species.

The inferred amino acid sequences of *Taenia solium* (cestode) and *Schistosoma haematobium* (trematode) RIOK-1 were truncated to match the catalytic kinase domain, for which several structures exist in the protein database (PDB) in their free and ligand-bound forms (*Homo sapiens* RIOK-1, *Archaeoglobus fulgidus* RIOK-1 and *Archaeoglobus fulgidus* RIOK-2). For all known RIOKs, the N-lobe, hinge region and C-lobe that form the minimum kinase domain are predicted to be crucial for kinase/ATPase activity^11^,^30^,^31^,^32^, and the number and location of their secondary structure elements are conserved^16^. The flexible loop, a characteristic feature of RIOKs^32^,^33^ comprising a sequence of ~20 amino acids, is located between the end of the P-loop and the adjacent α-helix. For flatworm RIOK-1 proteins, the conserved sequence pattern, based on four cestode and four trematode sequences is: T-S-I-x-x-P-F-K-S-x-K-Y-V-x-x-G-D-F-R (Figure 5).

Three-dimensional models of the cestode and trematode RIOKs (with exception of *Tsol*-RIOK-2), and *Hsap*-RIOK-2, were produced by threading and iterative template fragment assembly, followed by gene ontology analysis. For *Tsol*-RIOK-2, a comparative modelling approach guided by a structure-based sequence alignment was chosen and three-dimensional model generated using *Aful*-RIOK-2 as a template.

**Figure 6:**
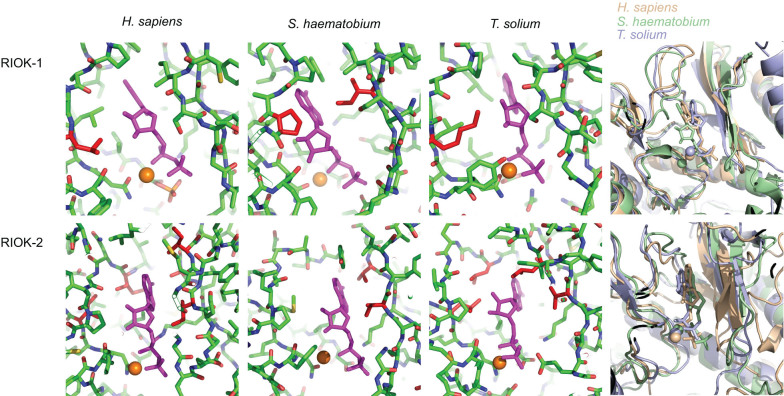
Comparison of the nucleotide binding sites in the catalytic domain of RIOK-1 (top row) and RIOK-2 (bottom row). In both rows, the last image shows a superposition of the nucleotide binding sites of human, cestode and trematode RIOKs. The first three images show views of the nucleotide binding sites for the individual RIOKs in the same orientation. Here, protein residues are rendered as atomic stick model (green: C, blue: N, red: O), the bound ADP molecule is shown in magenta, and the magnesium ion in orange. Variable amino acid residues among the three species are highlighted in red.

In order to structurally appraise the nucleotide-binding sites of flatworm RIOKs, Mg-ADP was manually docked into the sites of all RIOK models produced using the experimental structure of *Hsap*-RIOK-1 in complex with ADP as a template^11^. As expected, there was a high level of conservation when the nucleotide binding sites of flatworm RIOKs were compared with those of the human host (Supplementary Data 6 and Figure 6). In particular, the pivotal residue coordinating the magnesium ion (Asn329 in *Hsap*-RIOK-1) is strictly conserved among the six proteins studied. The binding poses shown in Figure 6 result from manual docking into modelled structures and are not refined. However, despite these approximations, very similar binding modes of Mg-ADP in the RIOKs of the three species are expected. The relative orientation and conformation of the modelled ADP molecules are immediately obvious when comparing the three panels in Figure 6 for either RIOK-1 or RIOK-2. Among the three species, the RIOK-2 nucleotide binding sites show few conservative amino acid substitutions (Supplementary Data 7 and Figure 6) and can thus be expected to engage in highly similar interactions with bound ligands. In contrast, a comparison of RIOK-1 models shows that only two positions in the nucleotide binding sites relate to amino acid variations: a conservative variation at V194, and a non-conservative variation at L289 (Supplementary Data 7 and Figure 6).

## Discussion

Based on an analysis of existing genomes and transcriptomes, we identified *riok-1*, *-2* and *-3* in five early metazoans, such as sponges and cnidarians, in five chordates and in ten ecdysozoans (including nematodes, insects and crustaceans). The presence of *riok-3* in molluscs and annelids but its absence from flatworms indicates that the loss of *riok-3* is unique to flatworms. A detailed analysis of representatives (n = 25) of the Phylum Platyhelminthes showed that *riok-3* has been lost from both parasitic and free-living flatworms. This information shows that the *riok-3* gene loss from flatworms is not related to a parasitic life style, but rather to the unique biology and/or physiology of flatworms. The present study suggests a role for RIOK-3 in ribosomal biosynthesis, and a co-localization and pull-down study of *Hsap*-RIOK-3 has indicated that RIOK-3 is a component of the small ribosomal unit (40S) biosynthesis complex, and is closely linked to ribosomal proteins ENP-1 and LTV-1^14^. Loss-of-gene function studies in *Caenorhabditis elegans* and *Drosophila melanogaster* have shown that, unlike *riok-1* and *riok-2*, *riok-3* is not essential for survival and development, but silencing of this kinase/ATPase does reduce the maturation rate of the small ribosomal 40S subunit^10^. Based on high sequence similarity in the kinase core-regions between the RIOK-1 and RIOK-3^32^*,* *riok-3* most likely originated from a *riok-1* gene-duplication event. We hypothesize that this gene duplication event has provided metazoan organisms with the capacity to increase ribosome maturation efficiency, allowing for time efficient protein production and cell cycle progression. Therefore, metazoans expressing RIOK-3 are likely able to increase their protein production capability, providing an evolutionary advantage. However, the question arises as to whether the loss of *riok-3*/RIOK-3 from flatworms has an impact on ribosomal biosynthesis and overall fitness.

Flatworms, such as the free-living planarian *Schmidtea mediterranea* are amongst the simplest animals with bilateral symmetry and tissues with distinct organs, and are known for their ability to “degrow” (reduce cell numbers and reabsorb reproductive organs) when food is scarce and to regenerate entire body parts^34^,^35^. This remarkable plasticity is driven by a small number of adult stem cells, called neoblasts - the only planarian flatworm cells that can divide to replace cell loss^36^. Recently, such neoblasts have been described in non-planarian, parasitic flatworms, such as *Echinococcus multilocularis*^37^,^38^. Some evidence also suggests that somatic cells of some flatworms do not undergo cell division^34^,^38^. This apparent lack of cell division in somatic cells is reflected in the reported loss of NIMA-related kinases (NEKs), a family of protein kinases known to play a key role in cell cycle progression^29^, and would result in a reduced protein production pressure on flatworm somatic cells. Therefore, we propose that the loss of the *riok-3* gene from flatworms and the likely reduction in ribosomal maturation efficiency have a limited impact on the non-dividing somatic cells of flatworms, not reducing flatworm fitness. To further support this proposal, we studied the presence/absence of RIOK-3-associating proteins in the ribosomal biosynthesis pathway. One would expect the loss of *riok-3* in flatworms to coincide with a loss of other SSU proteins. We identified all 30 ribosomal and seven associating SSU proteins in the late ribosomal biosynthesis pathway of the flatworms investigated here. The conservation of the SSU associated proteins among these flatworms, despite the loss of RIOK-3, raises questions as to the primary functional role of RIOK-3. This kinase has also been proposed to be involved in the regulation of NF-κB signalling, which is part of the TNF-R1 pathway^12^. In vertebrates, caspase-10 is one of the initiator enzymes downstream of TNF-R1, and is crucial for the activation of both apoptosis and the NF-κB signalling pathway^39^. Caspase-10 activates the apoptosis pathway through its proteolytic domain, while it activates the NF-κB pathway via its N-terminal death effector domains (DED)^40^. In the NF-κB pathway, the caspase-10 DED domains interact with specific kinases (RIP and NIK), independently of the proteolytic activity of caspase-10, and lead to cell growth and cell differentiation, an opposite response to the apoptosis pathway^40^. The molecular basis for cross-talk between these two pathways is still not well understood. In vertebrates, RIOK-3 has been shown to inhibit the NF-κB pathway, by competing with the kinases RIPK and NIK for the caspase-10 DED domains, independent of its ATPase activity^12^. Lee et al.^41^ suggested that the TNF-R1 pathway in flatworms has been lost and that apoptosis is limited to the intrinsic or stress pathway. A bioinformatic search showed the absence of caspase-10 orthologs from the flatworm genomes and transcriptomes studied here, suggesting that the loss of RIOK-3 is linked to those of caspase-10 and/or its target DED domains. In addition to the losses of RIOK-3 and caspase-10, we did not find most other upstream and downstream components of either the apoptosis or NF-κB pathway (Figure 4). However, we did identify TNF-R1 orthologs in flatworms, but these receptors are known to lack dead domains essential for intracellular signalling, supporting the loss of functionality of the extrinsic apoptosis and caspase-10-induced NF-κB pathways in flatworms^42^. Based on this analysis, we suggest that the loss of caspase-10 and its associating proteins represent a driving force for the loss of the *riok-3* gene from flatworms. In addition, the conservation of all SSU components among flatworms suggests that the regulation of cell death and cell growth through the TNF-R1 pathway represents the primary functional role for RIOK-3 rather than of ribosomal biosynthesis.

The *riok* genes are receiving increased attention because of their potential as drug targets for anti-cancer and anti-infective compounds^10^,^15^,^16^,^43^. This is particularly true for *riok-1* and *riok-2*, for which loss-of-gene-function studies have shown their essentiality and a likely involvement in vital biological processes, such as ribosomal biosynthesis, chromosome stability and/or cell cycle progression^1^,^2^,^3^,^4^,^5^,^6^,^7^,^8^,^9^,^10^,^11^. We showed that the *riok-1* and *riok-2* genes are present and transcribed in 52 metazoans studied here, including 25 flatworms representing trematodes and cestodes. The variability in the observed *riok* gene structures of four trematodes and four cestodes (exon numbers ranging from 3 to 7, and gene lengths ranging from 1,618 to 14,971 bp) is significantly lower compared with that previously reported for nine nematode species^16^. This variability in gene structure is not related to genome size of species. However, we did observe a positive relationship between the *riok* gene compactness and the respective evolutionary position of a flatworm species, suggesting relative conservation in *rio*k structure within the trematode and cestode groups.

Various classes of compounds, such as pyrazinoisoquinolines (praziquantel), benzimidazoles (triclabendazole), artemisinins (artesunate), synthetic trioxolanes (closantel) and synthetic peroxides^44^,^45^,^46^,^47^,^48^, are used to treat parasitic flatworm infections in humans or animals, but some are ineffective against particular developmental stages of some worms^45^,^47^, and others are limited by a narrow spectrum of activity and/or emergent drug resistance^45^,^47^. Therefore, there is a need to work toward new and improved drugs against flatworms based on an understanding of their molecular biology. In this context, it is critical to target gene products that are transcribed or expressed in suitably druggable developmental stages of these worms, as is true of RIOKs. The parasitic trematode *Schistosoma haematobium* displays consistent and moderate transcription levels of the *riok-1* and *riok-2* genes among developmental stages and between sexes^27^. A similar transcriptional profile is reported for three species of parasitic nematodes (*Haemonchus contortus*, *Brugia malayi* and *Ascaris* *suum*)^16^ and in the vinegar fly, *Drosophila melanogaster*^49^. The constitutive and moderate *riok* transcription levels observed across parasitic trematodes, nematodes and insects suggest that RIOKs perform house-keeping functions. This finding agrees with their predicted roles in essential pathways, such as ribosome biosynthesis and cell cycle progression^1^,^2^,^3^,^4^,^5^,^6^,^7^,^8^,^9^,^10^,^11^.

In the absence of crystal structures of RIOK proteins of flatworms, a molecular modelling approach was employed to compare nucleotide binding sites between parasite and mammalian RIOKs. Since these sites are considered druggable, they are targeted by the majority of current kinase inhibitors for therapeutic uses^50^. The topologies of these binding sites are very similar when compared among different kinases, and the problem of selectivity arises when designing inhibitors specifically against pathogen RIOKs. Selectivity not only relates to a distinction between parasite and host RIOK sites, but also nucleotide binding sites in any of the >500 human kinases. As a first step in this direction, the present study aimed to appraise any suitable variation in the nucleotide binding sites in RIOKs between flatworms (cestodes and trematodes) and a mammal (human). A promising lead may be the residue in position of L289 of human RIOK-1, which is P197 in *Schistosoma haematobium* and K187 in *Taenia solium*. The introduction of a basic residue in RIOK-1 of all cestodes and the space alteration of the nucleotide-binding site in orthologs in the case of trematodes are points of interest for the design of ligands that specifically target RIOK-1 proteins of flatworms, as opposed to those of the host.

## Methods

### Gene prediction and identification

From public databases, we extracted genomic, transcriptomic and protein datasets for the identification, isolation and curation of full-length *riok* genes from the trematodes *Clonorchis sinensis* (GenBank Assembly ID: GCA_000236345.1)*, Opisthorchis viverrini* (GenBank Assembly ID: GCA_000236345.1)*, Schistosoma haematobium* (NCBI BioProject ID: PRJNA78265), *Schistosoma mansoni* (GenBank Assembly ID: GCA_000237925.2) and *Schistosoma japonicum* (GenBank Assembly ID: GCA_000151775.1), and the cestodes *Echinococcus multilocularis* (GenBank Assembly ID: GCA_000469785.1), *Echinococcus granulosus* (GenBank Assembly ID: GCA_000524195.1), *Taenia* *solium* (NCBI BioProject ID: PRJNA183343) and *Hymenolepis microstoma* (GenBank Assembly ID: GCA_000469805.1). Using transcripts inferred from partial amino acid sequences of RIOK proteins, *riok* genes were located in individual genomes and extracted using the programs BLAT^51^ and Exonerate^52^. Transcription levels were then established by identifying corresponding *riok* transcripts in individual transcriptomes; each gene was manually curated to verify its full length. Using a web-based application (http://wormweb.org/exonintron; accessed: 04/11/2014), gene structures were displayed. The nomenclature of the *riok* gene family is based on a previous study^16^. Genes/proteins were named according to an abbreviation of the binomial species name, followed by the gene and the gene number. For example, *riok-1* of *S. haematobium* is called *Shae-riok-1*, and the encoded protein is *Shae-*RIOK-1.

### Phylogenetic analysis of flatworm RIOKs

HMM profiles for RIOK-1, -2 and -3 were derived from the databases NCBI homologene (http://www.ncbi.nlm.nih.gov/homologene; accessed: 04/11/2014) and KEGG (http://www.genome.jp/kegg/pathway.html; accessed: 04/11/2014). Through HMM scan searches of publicly available draft genomes of flatworms, available via the Wellcome Trust Sanger Institute (http://www.ebi.ac.uk/ena/data/view/PRJEB2709; accessed: 04/11/2014), we identified additional RIOKs in 11 trematode and 8 cestode species. Searches were also conducted to establish the presence or absence of *riok-1*, *riok-2* and *riok-3* genes in/from the genomes or transcriptomes of 44 distinct species of cnidarians, poriferans, chordates, insects, crustaceans, molluscs, annelids and plathyhelmiths. Subsequently, phylogenetic analysis of 50 manually curated and aligned RIOK amino acid sequences representing flatworms was conducted using Bayesian inference, employing the program MrBayes 3.1.2 (http://mrbayes.csit.fsu.edu/index.php; accessed: 04/11/2014). Posterior probabilities (pp) were calculated using 2,000,000 generations and the last 25% of these generations were used for phylogenetic analysis, employing four simultaneous tree-building chains, saving every 100^th^ tree. For each of the RIOK subfamilies found, a phylogenetic analysis of amino acid sequence data for the minimum kinase domain was conducted. RIOK sequences from *Crassotrea gigas* (a mollusc; GenBank Assembly ID: GCA_000297895) were used as outgroups; evolutionary distances were calculated using the toolkit BioPhylo^53^. Structural characteristics of individual genes were established (e.g., intron length, coding length, gene length, number of exons and genome size). General linear modelling in R was used to detect any relationships between evolutionary distance and gene structural characteristics for each RIOK subfamily.

### Structural analysis of flatworm and host RIOK-1 proteins

Structure-based amino acid sequence alignments were generated using the program SBAL^54^, and conserved and novel domains identified. The structure of *Hsap*-RIOK-1 was studied using the crystal structure deposited in the PDB (accession code 4otp)^11^. Three-dimensional models were built for truncated RIOK proteins from representative flatworms (RIOK-1 of *Taenia solium*, and RIOK-1 and RIOK-2 of *Schistosoma haematobium*) and their host species (RIOK-2 of *H. sapiens*), using the threading and iterative template fragment assembly approach, implemented in I-TASSER^55^. Models with a positive C-score (i.e. confidence score for estimating the quality of the predicted model; ranging between -5 and 2) and the highest TM-score (TeMplate quality-score of the generated 3D model and a known RIOK crystal structure) were selected. For *Tsol*-RIOK-2 (*Taenia solium*), a homology model was generated using the program MODELLER^56^ employing the crystal structure of *Aful*-RIOK-2 (*Archaeoglobus fulgidus*; PDB accession code 1zar) as a template. Briefly, a secondary structure-based amino acid sequence alignment of *Tsol*-RIOK-2 and *Aful*-RIOK-2 was prepared using SBAL^54^ and used to guide comparative modelling calculations. Twenty independent models were calculated, and that with the lowest energy was selected. All models were superimposed on the structure of *Hsap*-RIOK-1, and ADP docked into the five modelled structures by manual rigid-body docking using the program Coot^57^. An appraisal of the nucleotide binding site and possible protein-ligand interactions was undertaken by visual inspection of structures in Coot as well as distance analyses using in-house scripts (AH) from PCSB software^58^. Sequences of domains that displayed differences in the alignment between parasitic flatworms and human RIOK-1s were compared using MUSCLE-guided alignments^59^ and displayed using the program WebLogo^60^.

## Supplementary Material

Supplementary Information Supplementary Data

## Figures and Tables

**Table 1:** Features of the *riok* genes of eight parasitic flatworms representing the trematodes and cestodes, the features of these genes, and their exons and coding lengths

Flatworm group	Species	*riok* gene name	Gene length (bp)	Number of exons	Coding length (bp)
Trematodes	*Schistosoma haematobium*	*Shae-riok-1*	14971	3	1419
		*Shae-riok-2*	8734	6	1413
	*Schistosoma mansoni*	*Sman-riok-1*	10211	3	1410
		*Sman-riok-2*	8005	6	1413
	*Clonorchis sinensis*	*Csin-riok-1*	2242	3	1359
		*Csin-riok-2*	9820	7	1401
	*Opisthorchis viverrini*	*Oviv-riok-1*	2262	3	1410
		*Oviv-riok-2*	6741	7	1428
Cestodes	*Echinococcus multilocularis*	*Emul-riok-1*	2025	4	1410
		*Emul-riok-2*	4848	6	1578
	*Echinococcus granulosus*	*Egra-riok-1*	2021	4	1407
		*Egra-riok-2*	4852	6	1578
	*Hymenolepis microstoma*	*Hmic-riok-1*	3392	4	1437
		*Hmic-riok-2*	2977	6	1542
	*Taenia solium*	*Tsol-riok-1*	1989	4	1410
		*Tsol-riok-2*	1681	3	1488
