# Supplementary material for: Flatworms have lost the right open reading frame kinase 3 gene during evolution
Source: Sci Rep. 2015 May 15;5:9417. doi: 10.1038/srep09417 (PMC4894443; doi:10.1038/srep09417)
Supplement: Supplementary Information — Supplementary Data [file srep09417-s1.docx]

**Supplementary Data 1.** Flatworm species, abbreviated names and availability of genomic and/or transcriptomic data sets used for the extraction RIOK protein sequences for subsequent phylogenetic analyses. Published genomic data are listed in Supplementary Date1. Unpublished genomic data were from a database at the Wellcome Sanger Institute (<http://www.sanger.ac.uk/research/initiatives/globalhealth/research/helminthgenomes/>).

| Flatworm group | Species | Abbreviation | Published genome | Unpublished genome | Transcriptome |
| --- | --- | --- | --- | --- | --- |
| Cestoda | *Taenia taeniaeformis* | *Ttae* |  | ✔ |  |
|  | *Taenia solium* | *Tsol* | ✔ |  | ✔ |
|  | *Taenia asiatica* | *Tasi* |  | ✔ |  |
|  | *Echinococcus granulosus* | *Egra* | ✔ |  | ✔ |
|  | *Echinococcus multilocularis* | *Emul* | ✔ |  | ✔ |
|  | *Hymenolepis diminuta* | *Hdim* |  | ✔ |  |
|  | *Hymenolepis microstomata* | *Hmic* | ✔ |  | ✔ |
|  | *Hymenolepis nana* | *Hnan* |  | ✔ |  |
|  | *Mesocestoides corti* | *Mcor* |  | ✔ |  |
| Trematoda | *Opisthorchis viverrini* | *Oviv* | ✔ |  | ✔ |
|  | *Clonorchis sinensis* | *Csin* | ✔ |  | ✔ |
|  | *Facioloides magna* | *Fmag* |  |  | ✔ |
|  | *Faciola hepatica* | *Fhep* |  |  | ✔ |
|  | *Schistosoma haematobium* | *Shae* | ✔ |  | ✔ |
|  | *Schistosoma mansoni* | *Sman* | ✔ |  | ✔ |
|  | *Schistosoma margrebowiei* | *Smar* |  | ✔ |  |
|  | *Schistosoma intercalatum* | *Sint* |  | ✔ |  |
|  | *Schistosoma bovis* | *Sbov* |  | ✔ |  |
|  | *Schistosoma curassoni* | *Scur* |  | ✔ |  |
|  | *Schistosoma guineenssis* | *Sgui* |  | ✔ |  |
|  | *Schistosoma rodhaini* | *Srod* |  | ✔ |  |
|  | *Schistosoma mattheei* | *Smat* |  | ✔ |  |
|  | *Schistosoma turkestanicum* | *Stur* |  | ✔ |  |

**Supplementary Data 2**. Nexus file of amino acid sequence data for RIOK-1 used for the phylogenetic analysis.

#NEXUS

[TITLE: Written by EMBOSS 21/05/14]

begin data;

dimensions ntax=23 nchar=330;

format interleave datatype=protein missing=X gap=-;

matrix

Cgi_RIOK-1 KDRATAEQVMDPRTRMILFKFLQRGLIAEINGCISTKEANVYHATTQQGD

Hdim_RIOK1 GDRATTNHALDKRTIFIIFKMIHQGDFDEINGCISTKEANVYHAIS-RGD

Hmic_RIOK-1 GDRATTDHALDKRTVFIIFKMIHQGDFDEINGCISTKEANVYHAIS-RGD

Hna_RIOK1 ADRATTDHALDKRTVFIIFKMIHQGDFDKINGCISTKEANVYHAIS-NGD

Tta_RIOK1 GDRATTEHALDKRSVAILYKMMSQGEFAVINGCISTKEANVYHAIN-KGD

Egra_RIOK-1 GDRATTDHALDKRSVAILYKMMSQGEFAVINGCISTKEANVYHAIG-KSD

Emul_RIOK-1 GDRATTDHALDKRSVAILYKMMSQGEFAVINGCISTKEANVYHAIG-KGD

Tsol_RIOK-1 GDRATTDHALDKRSVAILYKMMSQGEFDAINGCISTKEANVYHAIN-KGD

Tas_RIOK1 GDRATTDHALDKRSVAILYKMMSQGEFDAINGCISTKEANVYHAIN-KGD

Mco_RIOK1 SDRATTDHALDRRSCAILYKMMSQGVYDEINGCISTKEANVYHAVG-NGD

Sro-RIOK-1 SDKATTDHALDRRSCSILFKMMNQEIFSEVNGCISTKEANIYHVKNKNVD

Sma-RIOK-1 SDKATTDHALDRRSCSILFKMMNQEIFSEVNGCISTKEANIYHVKNKNVD

Sbo-RIOK-1 SDKATTDHALDRRSCSILFKMMNQEIFSEVNGCISTKEANIYHVKNKNVD

Sha-RIOK-1 SDKATTDHALDRRSCSILFKMMNQEIFSEVNGCISTKEANIYHVKNKNVD

Sgu-RIOK-1 SDKATTDHALDRRSCSILFKMMNQEIFSEVNGCISTKEANIYHVKNKNVD

Scu-RIOK-1 SDKATTDHALDRRSCSILFKMMNQEIFSEVNGCISTKEANIYHVKNKNVD

Smar-RIOK-1 SDKATTDHALDRRSCSILFKMMNQEIFSEVNGCISTKEANIYHVKNKNVD

Sint-RIOK-1 SDKATTDHALDRRSCSILFKMMNQEIFSEVNGCISTKEANIYHVKNKNVD

Ovi-RIOK-1 SDRATTDHALDRRSRAILFKMMNQEIFTEINGCISTKEANIYHVLDKNTD

Csi-RIOK-1 SDRATTDHALDRRSRAILFKMMNQEIFTEINGCISTKEANIYHVLDKNTD

Fma-RIOK-1 SDRATTDHALDRRSRAILFKMMNQEIFSEINGCISTKEANIYHVIDKNTD

Fhe-RIOK-1 SDRATTDHALDRRSRAILFKMMNQEIFSEINGCISTKEANIYHVIDKNTD

Fgi-RIOK-1 SDRATTDHALDRRSRAILFKMMNQEIFSEINGCISTKEANIYHVIDKNTD

Cgi_RIOK-1 RAIKVYKTSILVFKDRDKYVTGEFRFRHGYCKHNPRKMVRTWAEKEMRNL

Hdim_RIOK1 LAIKLHMTSKLSFKARNKYVQGDFRMRHGYSTCSSWKLVSKWAEKEYRNL

Hmic_RIOK-1 LALKLHMTSKLAFKARNKYVQGDFRMRHGYSTCSSWKLVSKWAEKEYRNL

Hna_RIOK1 LALKLHMTSKLSFKARNKYVQGDFRMRHGYSTCSSWKLVSKWAEKEYRNL

Tta_RIOK1 LAIKVYMTSILPFKSRSKYVEGDFRMRHGYSTCSSWRLVSKWAEKEYRNL

Egra_RIOK-1 LAIKVYMTSILPFKSRSKYVEGDFRMRHGYSTCSSWRLVSKWAEKEYRNL

Emul_RIOK-1 LAIKVYMTSILSFKSRSKYVEGDFRMRHGYSTCSSWRLVSKWAEKEYRNL

Tsol_RIOK-1 IAIKVYMTSILPFKSRSKYVEGDFRMRHGYSTCSSWRLVSKWAEKEYRNL

Tas_RIOK1 IAIKVYMTSILPFKSRSKYVEGDFRMRHGYSTCSSWKLVSKWAEKEYRNL

Mco_RIOK1 MAIKLYMTAILPFKSRSKYVEGDFRMRRGYSTCSSWKLVSKWAEKEYRNL

Sro-RIOK-1 FAIKVYMTSIMPFKSRDKYVKGDFRMRHGYSKSTSWKLVCKWSEKEYRNL

Sma-RIOK-1 FAIKVYMTSIMPFKSRDKYVKGDFRMRHGYSKSTSWKLVCKWSEKEYRNL

Sbo-RIOK-1 LAIKVYMTSIMPFKSRDKYVKGDFRMRHGYSKSTSWKLVCKWSEKEYRNL

Sha-RIOK-1 LAIKVYMTSIMPFKSRDKYVKGDFRMRHGYSKSTSWKLVCKWSEKEYRNL

Sgu-RIOK-1 LAIKVYMTSIMPFKSRDKYVKGDFRMRHGYSKSTSWKLVCKWSEKEYRNL

Scu-RIOK-1 LAIKVYMTSIMPFKSRDKYVKGDFRMRHGYSKSTSWKLVCKWSEKEYRNL

Smar-RIOK-1 FAIKVYMTSIMPFKSRDKYVKGDFRMRHGYSKSTSWKLVCKWSEKEYRNL

Sint-RIOK-1 FAIKVYMTSIMPFKSRDKYVKGDFRMRHGYSKSTSWKLVCKWSEKEYRNL

Ovi-RIOK-1 LAVKVYMTSIMPFKSRDKYVKGDFRMRHGYSKATSWKLVSKWAEKEYRNL

Csi-RIOK-1 LAIKVYMTSIMPFKSRDKYVKGDFRMRHGYSKATSWKLVSKWAEKEYRNL

Fma-RIOK-1 LAIKVYMTSVMPFKCRDKYVKGDFRMRHGYSKATSWKLVSKWTEKEYRNL

Fhe-RIOK-1 LAIKVYMTSIMPFKCRDKYVKGDFRMRHGYSKATSWKLVSKWTEKEYRNL

Fgi-RIOK-1 LAIKVYMTSIMPFKCRDKYVKGDFRMRHGYSKATSWKLVSKWTEKEYRNL

Cgi_RIOK-1 SRMYQAG-LPCPEPIFLKSHVLVMRFIGTDGWPAPLLKDCRELYLECIHI

Hdim_RIOK1 VRIDKAGSIPSPRPLKLKGVLVLMTLIGKNGLPAPKLKDVPVIYRQVLEN

Hmic_RIOK-1 IRIEKAGSIPSPRPLKLKGVLVLMTLIGKNGLPAPKLKDVPVLYRQVLEN

Hna_RIOK1 IRIEKAGSIPSPRPLKLKGVLVLMTLIGKNGLPAPKLKDVPLLYRQVLEN

Tta_RIOK1 LRINRAGSIPAPAPVKLKGVVLLMTLIGKDGYPAPKLKDVPAIYRQILQN

Egra_RIOK-1 IRINKAGSIPTPLPIKLKGVVLLMTLIGKNGYPAPKLKDVSAIYRQILQN

Emul_RIOK-1 IRINKAGSIPTPLPIKLKGVVLLMTLIGKNGYPAPKLKDVSALYRQILQN

Tsol_RIOK-1 IRINKAGSIPAPLPIKLKGVVLLMTLIGKNGYPAPKLKDVSALYRQILQS

Tas_RIOK1 IRINKAGSIPAPLPIKLKGVVLLMTLIGKNGYPAPKLKDVSALYRQILQN

Mco_RIOK1 LRINMARTISAPVPIKLKGVVLLMTLIGKDGLPAPKLKDVPTLYRQILVD

Sro-RIOK-1 LRINQSGLISAPKPLRLKGVVLLMTFVGKDGIPAPKLKDVAALYFQVIHD

Sma-RIOK-1 LRINQSGLISAPKPLRLKGVVLLMTFVGKDGIPAPKLKDVASLYFQVIHD

Sbo-RIOK-1 LRINQSGLISAPKPLRLKGVVLLMTFVGKDGIPAPKLKDVAALYFQVIHD

Sha-RIOK-1 LRINQSGLISAPKPLRLKGVVLLMTFVGKDGIPAPKLKDVAALYFQVIHD

Sgu-RIOK-1 LRINQSGLISAPKPLRLKGVVLLMTFVGKDGIPAPKLKDVAALYFQVIHD

Scu-RIOK-1 LRINQSGLISAPKPLRLKGVVLLMTFVGKDGIPAPKLKDVAALYFQVIHD

Smar-RIOK-1 LRINQSGLISAPKPLRLKGVVLLMTFVGKDGIPAPKLKDVAALYFQVVHD

Sint-RIOK-1 LRINQSGLISAPKPLRLKGVVLLMTFVGKDGIPAPKLKDVAALYFQVIHD

Ovi-RIOK-1 IRIKQSGLIPCPTPLRLKGVVLLMSFIGKHGFPAPKLKDAPSLYAQVVND

Csi-RIOK-1 IRIKQSGLIPCPTPLRLKGVVLLMSFIGKNGFPAPKLKDAPSLYAQVVND

Fma-RIOK-1 IRIKQSGLIPCPTPLRLKGVVLLMSFVGKDGIPAPKLKDAPKLYAQVVND

Fhe-RIOK-1 IRINQSGLIPCPTPLRLKGVVLLMSFVGKNGIPAPKLKDAPRLYAQVVND

Fgi-RIOK-1 IRINQSGLIPCPTPLRLKGVVLLMSFVGKNGIPAPKLKDAPRLYAQVVND

Cgi_RIOK-1 IRTLYHTCRLIHADLSEFNMLYHDGGVYVIDVSQSVEHDHPCALEFLRKD

Hdim_RIOK1 VRTLFQKCRLVHGDLSEYNLLYMDGRAWMIDVSQAVEHECDQALELLRED

Hmic_RIOK-1 VRTLFQKCRLVHGDLSEYNLLYMDGRAWMIDVSQAVEHECDQALELLRED

Hna_RIOK1 VRTLFQKCRLVHGDLSEYNLLYMDGRAWMIDVSQAVEHECDQALELLRED

Tta_RIOK1 VRTLFQKCRLVHADLSEYNLLYMDGQAWFIDVSQAVEHECEQALEFLRKD

Egra_RIOK-1 VRTLFQKCRLVHADLSEYNLLYMDGQAWLIDVSQAVEHECEQALEFLRKD

Emul_RIOK-1 VRTLFQKCRLVHADLSEYNLLYMDGQAWLIDVSQAVEHECEQALEFLRKD

Tsol_RIOK-1 VRTLFQKCRLVHADLSEYNLLYMDGQAWLIDVSQAVEHECDQALEFLRKD

Tas_RIOK1 VRTLFQKCRLVHADLSEYNLLYMDGRAWLIDVSQAVEHECDQALEFLRKD

Mco_RIOK1 VRTLYQKCRLVHADLSEYNLLYMDGKAWMIDVSQAVEHEAPQALDFLRND

Sro-RIOK-1 IRTLFQKCRLVHADLSEYNLLYLDGKVWMIDVSQAVEHESPQALEYLRTD

Sma-RIOK-1 IRTLFQKCRLVHADLSEYNLLYLDGKVWMIDVSQAVEHESPQALEYLRTD

Sbo-RIOK-1 IRTLFQKCRLVHADLSEYNLLYLDGRVWMIDVSQAVEHESPQALEYLRAD

Sha-RIOK-1 IRTLFQKCRLVHADLSEYNLLYLDGRVWMIDVSQAVEHESPQALEYLRTD

Sgu-RIOK-1 IRTLFQKCRLVHADLSEYNLLYLDGRVWMIDVSQAVEHESPQALEYLRTD

Scu-RIOK-1 IRTLFQKCRLVHADLSEYNLLYLDGRVWMIDVSQAVEHESPQALEYLRTD

Smar-RIOK-1 IRTLFQKCRLVHADLSEYNLLYLDGRVWMIDVSQAVEHESPQALEYLRTD

Sint-RIOK-1 IRTLFQKCRLVHADLSEYNLLYLDGRVWMIDVSQAVEHESPQALEYLRTD

Ovi-RIOK-1 VRTLYQKCRLIHADLSEYNMLYMDGKAWMIDVSQAVEHESPQALDYLRTD

Csi-RIOK-1 VRTLFQKCRLIHADLSEYNMLYMDGKAWMIDVSQAVEHESPQALDYLRAD

Fma-RIOK-1 VRTLYQKCRLVHADLSEYNLLYMDDKVWMIDVSQSVEHESPQALDYLRSD

Fhe-RIOK-1 VRTLYQKCRLVHADLSEYNLLYMDDKVWMIDVSQSVEHESPQALDYLRSD

Fgi-RIOK-1 VRTLYQKCRLVHADLSEYNLLYMDDKVWMIDVSQSVEHESPQALDYLRSD

Cgi_RIOK-1 CTNVTEFFKKKNVSTLTVKELFDFVTDATITEDNID--YLEKVMTLASER

Hdim_RIOK1 CYNVNNFFRRQGVITLTLREFFEWVVDPTLPQEENES-YLDDFMKHAEER

Hmic_RIOK-1 CYNVNHFFRRQGVSTLTLREFFEWVVDPTLPQEENES-FLNAIMKRAEER

Hna_RIOK1 CYNVNHFFRRQGVSTLTLREFFEWVVDPTLPQEENES-FLDVIMKHAEER

Tta_RIOK1 CYNVNAFFRRQGVSTLTLREFFEWVVDPTLSEEVEEA-YLDRLLTRAETR

Egra_RIOK-1 CYNVNAFFRRQGVNTLTLREFFEWVVDPTLPQEGKEA-YLDTLLTRAQMR

Emul_RIOK-1 CYNVNAFFRRQGANTLTLREFFEWVVDPTLPEEGKEA-YLDTLLTRAQMR

Tsol_RIOK-1 CYNVNAFFRRQGVSTLTLREFFEWVVDPTLPEEVEEA-YLGTLLTRAEMR

Tas_RIOK1 CYNVNAFFRRQGVSTLTLREFFEWVVDPTLPEEVEEA-YLDTLLTRAEMR

Mco_RIOK1 CYNVNAFFRRQGVSTLTLREFFEWAVDPSLPQTEGPS----YLLALAEKR

Sro-RIOK-1 CHNINIFFRKQGVSTLTLRELFEWVVNPTLPAPDDASKCLMSLLREASIR

Sma-RIOK-1 CHNINIFFRKQGVSTLTLRELFEWVVNPTLPAPDDASKCLMSLLREASIR

Sbo-RIOK-1 CHNINIFFRKQGVSTLTLRELFEWVVNPSLPAPDDASKCLMSLLREASIR

Sha-RIOK-1 CHNINIFFRKQGVSTLTLRELFEWVVNPSLPAPDDASKCLMSLLREASIR

Sgu-RIOK-1 CHNINIFFRKQGVSTLTLRELFEWVVNPSLPAPDDASKCLMSLLREASIR

Scu-RIOK-1 CHNINIFFRKQGVSTLTLRELFEWVVNPSLPAPDDASKCLMSLLREASIR

Smar-RIOK-1 CHNINIFFRKQGVSTLTLRELFEWVVNPSLPAPDDASKCLMSLLREASIR

Sint-RIOK-1 CHNINIFFRKQGVSTLTLRELFEWVVNPSLPAPDDASKCLMSLLREASIR

Ovi-RIOK-1 CHNVNTFFRRQGVSTLTLREFFDWVVNPSLPQPDDPAQYLTRLLETAEER

Csi-RIOK-1 CHNVNNFFRRQGVSTLTLREFFDWVVNPSLPQPDDPAQ-LTRLLETAEER

Fma-RIOK-1 CYNVNTFFRKQGVTTLTLREFFEWVVNPSLPAPDDPAHYLQQLLQAAQER

Fhe-RIOK-1 CYNVNTFFRKQGVTTLTLREFFEWVVNPSLPAPDDPAHYLQQLLQAAQER

Fgi-RIOK-1 CYNVNTFFRKQGVTTLTLREFFEWVVNPSLPAPDDSAHYLQKLLQAAQER

Cgi_RIOK-1 STDDITEQQEEVFKHSFIPRNLDEVIDFERDVIMAKEG--QTEGMLYHTL

Hdim_RIOK1 GFNKTLEVEDDAFRSVYVPRRLEDVRRYVSDLKRLRAGLIKPEDLYYTAV

Hmic_RIOK-1 GFNETLDVEDDAFRRVYVPRRLEDVRRYVRDLKRLKAGIIKPEDLYYTAV

Hna_RIOK1 GFNETLNVEDDAFRRVYVPRRLEDVRRYVRDLKRLKTGIIKPEDLYYTAV

Tta_RIOK1 GFNRTLEIEDDAFRRVYVARRLEDVKRFFSDFKRLKMGLIKPEDLYYTAV

Egra_RIOK-1 GFNQTLEIEDDAFRRVYVARRLEDVKRFFSDFKRLKMGLIKPEDLYYTAV

Emul_RIOK-1 GFNQTLEIEDDAFRRVYVARRLEDVKRFFSDFKRLKMGLIKPEDLYYTAV

Tsol_RIOK-1 GFNQTLEIEDDAFRRVYVPRRLEDVKRFFSDFKRLKMGLIKPEDLYYTAV

Tas_RIOK1 GFNQTLEIEDDAFRRVYVPRRLEDVKRFFSDFKRLKMGLIKPEDLYYTAV

Mco_RIOK1 GHNQTLLTEDDAFRRVYVPRSLFEVKRFFQDFVRLKKGLIKPEDLYYTAV

Sro-RIOK-1 GLNETIEKEDEAFRYVHIPRNLSVSYPFVRDFLKIQRGQLSHSDIYYAAI

Sma-RIOK-1 GLNETIEKEDEAFRYVHIPRNLSVSYPFVRDFLKIQRGQLSHSDIYYAAI

Sbo-RIOK-1 GLNETIEKEDEAFRYVHIPRNLSVSYPFVRDFLKIQRGQLSHSDIYYAAI

Sha-RIOK-1 GLNETIEKEDEAFRYVHIPRNLSVSYPFVRDFLKIQRGQLSHSDIYYAAI

Sgu-RIOK-1 GLNETIEKEDEAFRYVHIPRNLSVSYPFVRDFLKIQRGQLSHSDIYYAAI

Scu-RIOK-1 GLNETIEKEDEAFRYVHIPRNLSVSYPFVRDFLKIQRGQLSHSDIYYAAI

Smar-RIOK-1 GLNETIEKEDEAFRYVHIPRNLSVSYPFVRDFLKIQRGQLSHSDIYYAAI

Sint-RIOK-1 GLNETIEKEDEAFRYVHIPRNLSVSYPFVRDFLKIQRGQLSHSDIYYAAI

Ovi-RIOK-1 GFNQTVEAEDNAFRFVHIPRNLSAVYPFVRDFLKMQRGLLSPDDVYYASV

Csi-RIOK-1 GFNQTVEAEDNAFRFVHIPRNLSAVYPFVRDFLKMQRGLLSPEDVYYASV

Fma-RIOK-1 GFNQTIEVEDSAFRYVHIPRNLQAAYPFVRDFLRLQTGKLTPSEVYYAAV

Fhe-RIOK-1 GFHETIEVEDSAFRYVHIPRNLQASYPFVRDFLRLQIGKLTPSEVYYAAV

Fgi-RIOK-1 GFNETIEVEDSAFRYVHIPRNLQASYPFVRDFLRLQIGKLTPSEVYYAAV

Cgi_RIOK-1 TGLQENLAEDRRLREYLSEIKKEISRERLE

Hdim_RIOK1 TGVKSGLPESKRARKRQEKIPKHVKKRATK

Hmic_RIOK-1 TGVKSGLPESKKARKRREKIPKHVKKRAAK

Hna_RIOK1 TGVKSGLPESKKARKRREKIPKHVKKRAAK

Tta_RIOK1 TGVRPELLASKRSRKRLTKIPKHVKKRAKK

Egra_RIOK-1 TGVRSGLLESKRSRKRLTKIPKHVKKRARK

Emul_RIOK-1 TGVRSGLLESKRSRKRLAKIPKHVKKRARK

Tsol_RIOK-1 TGVRSELLESKRSRKRLTKIPKHVKKRAKK

Tas_RIOK1 TGVRSELRESKRSRKRLTKIPKHVKKRAKK

Mco_RIOK1 TGVRSDLPGSKRTRKRQEKIPKSVKKRATK

Sro-RIOK-1 TGLKPDLPESRRNRKRKHKIPKYVKRRKIK

Sma-RIOK-1 TGLKPDLPESRRNRKRKHKIPKYVKRRKIK

Sbo-RIOK-1 TGLKPDLPESRRNRKRKHKIPKYIKRRKIK

Sha-RIOK-1 TGLKPDLPESRRNRKRKHKIPKYVKRRKIK

Sgu-RIOK-1 TGLKPDLPESRRNRKRKHKIPKYVKRRKIK

Scu-RIOK-1 TGLKPDLPESRRNRKRKHKIPKYIKRRKIK

Smar-RIOK-1 TGLKPDLPESRRNRKRKHKIPKYVKRRKIK

Sint-RIOK-1 TGLKPDLPESRRNRKRKHKIPKYVKRRKIK

Ovi-RIOK-1 SGMKQDLPASRKLRKRKTKIPKHVKRRRPK

Csi-RIOK-1 SGMKQDLPASRKLRKRKTKIPKHVKRRRPK

Fma-RIOK-1 SGMKQDLPTSRKLRKRKTKIPKHIKRHRTK

Fhe-RIOK-1 SGMKQDLPASRKLRKRKTKIPKHVKRHRTK

Fgi-RIOK-1 SGMKQDLPASRKLRKRKTKIPKHVKRHRTK

;

end;

begin mrbayes;

log start replace filename = mrbayes.log;

prset aamodelpr=mixed;

lset rates=invgamma;

prset ratepr=variable;

showmodel;

mcmc ngen=2000000 printfreq=10000 samplefreq=100

nchains=4 diagnfreq=1000

nruns=2 nperts=2;

sumt relburnin=yes burninfrac=0.25 contype=halfcompat;

sump relburnin=yes burninfrac=0.25;

log stop;

end;

**Supplementary Data 3.** Nexus file of amino acid sequence data for RIOK-2 used for the phylogenetic analysis.

#NEXUS

[TITLE: Written by EMBOSS 21/05/14]

begin data;

dimensions ntax=23 nchar=308;

format interleave datatype=protein missing=X gap=-;

matrix

Cgi_RIOK2 MGMKNHELVPSPLVASIAHLHHVLR-LNKHRLVAYERSGKRFEGYRLTVS

Hna_RIOK2 MGMKNHEFVPLDLVHKISKCTRLLRDLVPHGLLAYETDNKKYSGYRLTNL

Hdi_RIOK2 MGMKNHEFVPLDLVHKISKCTRLLRDLVPHGLLAYETDNKKYSGYRLTNL

Hmic_RIOK-2 MGMKNHEFVPLDLVHKISKCTRLLRDLVPHGLLAYETDNKKYSGYRLTNL

Mco_RIOK2 MGMKNHEFVPSDLVHKISRCARLLRDLVPHGLLAYETDNRKYSGYRLTNL

Tta_RIOK2 MGMKNHEFVPIDLVHKISRCSRLLRDLVPHGLLAYENDSRKYSGYRLTNL

Tsol_RIOK-2 MGMKNHEFVPLDLVHKISRCACLLRDLVPHGLLAYETDSRKYSGYRLTNL

Tas_RIOK2 MGMKNHEFVPLDLVHKISRCARLLRDLVPHGLLAYENDSRKYSGYRLTNL

Egra_RIOK-2 MGMKNHEFVPLDLVHKISRCARLLRDLVPHGLLAYEGDSRKYSGYRLTNL

Emul_RIOK-2 MGMKNHEFVPLDLVHKISRCARLLRDLVPHGLLAYESDSRKYSGYRLTNL

Stu_RIOK2 MGLKNHEVVPPELALKISHLRHLVQQLILNRLVAYETDNKHMKGYRLTNL

Sro_RIOK2 MGLKNHEVVPPELALKISHLRHLVQQLILNKLVAYETDNRHMKGYRLTNL

Sma_RIOK2 MGLKNHEVVPPELALKISHLRHLVQQLILNKLVAYETDNRHMKGYRLTNL

Sha_RIOK2 MGLKNHEVVPPELALKISHLRHLVQQLILNRLVAYETDNKHMKGYRLTNL

Smat_RIOK2 MGLKNHEVVPPELALKISHLRHLVQQLILNRLVAYETDNKHMKGYRLTNL

Scu_RIOK2 MGLKNHEVVPPELALKISHLRHLVQQLILNRLVAYETDNKHMKGYRLTNL

Sint_RIOK2 MGLKNHEVVPPELALKISHLRHLVQQLILNRLVAYETDNRHMKGYRLTNL

Smar_RIOK2 MGLKNHEVVPPELALKISHLRHLVQQLILNRLVAYETDNRHMKGYRLTNL

Fma-RIOK-2 MGLKNHEVVPAELALKISRCKRIVRQLIPNSLVAYEGDSKRISGYRLTNL

Fhe-RIOK-2 MGLKNHEVVPAELALKISRCKRIVRQLIPNSLVAYEGDSKRISGYRLTNL

Fgi-RIOK-2 MGLKNHEVVPAELALKISRCKRIVRQLIPNSLVAYEGDSKRISGYRLTNL

Ovi_RIOK2 MGMKNHEVVPLELAQKISRCKKLIKQLVSNSLVAYESDSRRVCGYRLTNL

Csi_RIOK2 MGMKNHEVVPLELAQKISRCKKLIKQLVSNSLVAYESDSRRVCGYRLTNL

Cgi_RIOK2 GYDYLALKALASRDVIYSLGNQIGVGKESDIYIIADDHQYALKLHRLGRT

Hna_RIOK2 GYDYLALHTLIKSGQICDLGSIIGVGKESDVYLAVAGENIVIKFHRLGRT

Hdi_RIOK2 GYDYLALHTLIKGGQICDLGSIIGVGKESDVYLAVAGETIVIKFHRLGRT

Hmic_RIOK-2 GYDYLALHTLIKSGQICDLGSIIGVGKESDVYLAVAGETIVIKFHRLGRT

Mco_RIOK2 GYDYLALHALIKSGQVIDLGSMIGVGKESDVYLAVAGDSIVIKFHRLGRT

Tta_RIOK2 GYDYLALNTLTKSGQVIDLGSMIGSGKESDVYLALAGDLIVIKFHRLGRT

Tsol_RIOK-2 GYDYLALHTLSKSGQVIDLGSMIGAGKESDVYLALAGEMIVIKFHRLGRT

Tas_RIOK2 GYDYLALHTLSKSGQVIDLGSMIGTGKESDVYLALAGEMIVIKFHRLGRT

Egra_RIOK-2 GYDYLALHTLTKSGQVIDLGSMIGAGKESDVYLAVAGDMIVIKFHRLGRT

Emul_RIOK-2 GYDYLALHALTKSGQVIDLGSMIGAGKESDVYLAVAGDMIVIKFHRLGRT

Stu_RIOK2 GYDYLALNQLFKSEQLTSLGTMIGAGKESDVYIAAAEDLVVVKFHRLGRT

Sro_RIOK2 GYDYLALNQLFKSEQLASLGTMIGAGKESDVYIATAGDAVVVKFHRLGRT

Sma_RIOK2 GYDYLALNQLFKSEQLASLGTMIGAGKESDVYIATAGDAVVVKFHRLGRT

Sha_RIOK2 GYDYLALNQFFKSEQLESLGTMIGAGKESDVYIAAAGDSVVVKFHRLGRT

Smat_RIOK2 GYDYLALNQFFKSEQLESLGTMIGAGKESDVYIAAAGDPVVVKFHRLGRT

Scu_RIOK2 GYDYLALNQFFKSEQLESLGTMIGAGKESDVYIAAAGDSVVVKFHRLGRT

Sint_RIOK2 GYDYLALNQFFKSEQLESLGTMIGAGKESDVYIAAAGDSVVVKFHRLGRT

Smar_RIOK2 GYDYLALNQFFKSEQLESLGTMIGAGKESDVYIAAAGDSVVVKFHRLGRT

Fma-RIOK-2 GYDYLALHQLFKSGQLADLGSMIGTGKESDVYIGVAGEPVVVKFHRLGRT

Fhe-RIOK-2 GYDYLALHQLFKSGQLADLGSMIGTGKESDVYIGVAGEPVVVKFHRLGRT

Fgi-RIOK-2 GYDYLALHQLFKSGQLADLGSMIGTGKESDVYIGVAGEPVVVKFHRLGRT

Ovi_RIOK2 GYDYLALHQLFNSGQLCDLRTMIGAGKESDVYLAIAGSPVVVKFHRLGRT

Csi_RIOK2 GYDYLALHQLFNSGQLCDLGTMIGAGKESDVYLAVAGSPVVVKFHRLGRT

Cgi_RIOK2 SFRQLKNKRDYHKHRNNVSWLYLSRLAAMKEYAYMKALYERKFPVPKPVD

Hna_RIOK2 SFRKVREKREYHQGRNTCSWLYLDRLAAKREYEMMQILYDDGLPVPCPLA

Hdi_RIOK2 SFRKVREKREYHQGRNTCSWLYLDRLAAKREYEMMKILYDDGLPVPSPLA

Hmic_RIOK-2 SFRKVREKREYHQGRNTCSWLYLDRLAAKREYEMMQILYDDGLPVPCPLA

Mco_RIOK2 SFRKVREKREYHQGRNTCSWLYLDRLAAKREFEMMQILYDYGLPVPCPLA

Tta_RIOK2 SFRKVREKREYHQHRNTCSWLYLDRLAAKREYEMMRMLYHHGLPVPCPLA

Tsol_RIOK-2 SFRKVREKREYHQHRNTCSWLYLDRLAARREFEMMQVLYGHGLPVPCPLA

Tas_RIOK2 SFRKVREKREYHQHRNTCSWLYLDRLAARREFEMMQMLYRHGLPVPCPLA

Egra_RIOK-2 SFRKVREKREYYQHRNTCSWLYLDRLAAKREFEMMRILYHHGLPVPCPLA

Emul_RIOK-2 SFRKVREKREYHQHRNTCSWLYLDRLAAKREFEMMRILYHHGLPVPCPLA

Stu_RIOK2 SFRKVKEKREYHQHRNNCSWLYLDRLASKREFIMMQSLWSNGIPVPIPYT

Sro_RIOK2 SFRKVKEKREYHQHRSSCSWLYLDRLASRREFVMMQSLRSKGVPVPIPYT

Sma_RIOK2 SFRKVKEKREYHQHRSSCSWLYLDRLASRREFVMMQSLRSKGIPVPIPYT

Sha_RIOK2 SFRKVKEKRDYHQHRSSCSWLYLDRLASRREFVMMQSLRSNGIAVPIPYT

Smat_RIOK2 SFRKVKEKRDYHQHRSSCSWLYLDRLASRREFVMMQSLRSNGIPVPIPYT

Scu_RIOK2 SFRKVKEKREYHQHRSSCSWLYLDRLASRREFVMMQSLRSNGIPVPIPYT

Sint_RIOK2 SFRKVKEKREYHQHRSSCSWLYLDRLASRREFVMMQSLRSNGIPVPIPYT

Smar_RIOK2 SFRKVKEKREYHQHRSSCSWLYLDRLASRREFVMMQSLRSNGIPVPIPYT

Fma-RIOK-2 SFRKVKEKREYHQHRNTCSWLYLDRLASQREFAMMKVLYDRGLPVPIPLA

Fhe-RIOK-2 SFRKVKEKREYHQHRNTCSWLYLDRLASQREFDMMKVLYNHGLPVPIPLA

Fgi-RIOK-2 SFRKVKEKREYHQHRNTCSWLYLDRLASQREFDMMKVLYDHGLPVPIPLA

Ovi_RIOK2 SFRKVKEKREYHQHRKACSWLYLDRLASSREFLMMKALHSHHVAVPQPLA

Csi_RIOK2 SFRKVKEKREYHQHRKACSWLYLDRLASSREFLMMKALHSHHVAVPQPLA

Cgi_RIOK2 FNRHAVVMELLSYHDCMELIVRLGNCGVIHGDFNEFNLMIDDEGNVTMID

Hna_RIOK2 NNRNAVVMSLISYSQAKEILAKITAEGLVHGDFNEFNLLVSGLAKLVLID

Hdi_RIOK2 NNRNAVVMSLVPYSQAREILAKITAEGLVHGDFNEFNLLVSGLAKLVLID

Hmic_RIOK-2 NNRNAVVMSLIPYSQAKEILAKITAEGLVHGDFNEFNLLISGLAKLVLID

Mco_RIOK2 NNRNAVVMSLVSYAQAVEILEKITSNGLIHGDFNEFNLLIGGLVQLILID

Tta_RIOK2 NNRNAVVMSLLMYAQAVDILDNITRNGLVHGDFNEFNLLVHGLPKLFLID

Tsol_RIOK-2 NNRNAVVMSLLAYSQAVDILTTITRNGLIHGDFNEFNLLVHGLTKLILID

Tas_RIOK2 NNRNAVVMSLLAYSQAVDILTTITRNGLIHGDFNEFNLLVYGLAKLILID

Egra_RIOK-2 NNRNAVVMSFLAYTQAADILSTITRNGLIHGDFNEFNLLVHGLAKLILID

Emul_RIOK-2 NNRNAVVMSFLAYTQAVDILSTITRNGLIHGDFNEFNLLVHGLAKLILID

Stu_RIOK2 HNRNAVVMSYVAYYQAKEILERVASLGLVHGDFNEFNLMVSDLDKLVLID

Sro_RIOK2 HNRNAVVMSYVAYYQAKDILERVVSLGLVHGDLNEFNLMVSDLDKLVLID

Sma_RIOK2 HNRNAVVMSYVAYYQAKDILERVASLGLVHGDLNEFNLMVSDLDKLVLID

Sha_RIOK2 HNRNAVVMSYIAYYQAKNILERIASLGLVHGDLNEFNLMVSDLDKLVLID

Smat_RIOK2 HNRNAVVMSYIAYYQAKNILERIASLGLVHGDLNEFNLMVSDLDKLVLID

Scu_RIOK2 HNRNAVVMSYIAYYQAKNILERIASLGLVHGDLNEFNLMVSDLDKLILID

Sint_RIOK2 HNRNAVVMSYIAYYQAKNILERIASLGLVHGDLNEFNLMVSDLDKLVLID

Smar_RIOK2 HNRNAVVMSYIAYYQAKNILERIASLGLVHGDLNEFNLMVSDLDKLVLID

Fma-RIOK-2 HNRNAVMMSYIAYMQARDMLHKIASIGLIHGDFNEFNLMVVGLSKLVLID

Fhe-RIOK-2 HNRNAVMMSYISYMQARDMLHKIASIGLIHGDFNEFNLMVVGLSKLVLID

Fgi-RIOK-2 HNRNAVMMSYISYMQARDMLHKIASIGLIHGDFNEFNLMVVGLSKLVLID

Ovi_RIOK2 HNRNAVVMSYVAYSQAREMLQKIASLGLIHGDFNEFNLMVVGLGKLVLID

Csi_RIOK2 HNRNAVVMSYVAYSQAREMLQKIASLGLIHGDFNEFNLMVVGLGKLVLID

Cgi_RIOK2 FPQMVSTSHYNAEFDRDVTCIRDFFARRFNYESELYP--KFSDLRRDDDL

Hna_RIOK2 FPQMISRDHWSAQYERDLDGILSFFGKFLDIAPDEMPPRNLNEIPRTGNM

Hdi_RIOK2 FPQMISRDHWTAQYERDLDGIVSFFGKFLDIAPDEMPLRNLKEIPRTGYM

Hmic_RIOK-2 FPQMISRDHWTAQYERDLDGILSFFGKFLDIAPDEMPPRNLKEIPRTGYM

Mco_RIOK2 FPQMISRDHRNAQYERDLNGIVGFFDKFLELDPTNIPPKSLLDIPRTGSM

Tta_RIOK2 FPQMISRNHPTAQYERDLNGIISFFSRFLEISPADTPPRSLTDIPRTGHM

Tsol_RIOK-2 FPQMISRDHRTAQYERDLNGIVSFFSRYLEIPPADIPPRSLADVPRTGNM

Tas_RIOK2 FPQMISRDHRTAQYERDLNGIVSFFSRYLEISPADIPPRSLADVPRTGNM

Egra_RIOK-2 FPQMISRDHRTAQYERDLNGIVNFFSRFLEIPPTDVPPRSLADVPRTGNM

Emul_RIOK-2 FPQMISRDHRTAQYERDLNGIVNFFSRFLEIPPTDVPPRSLADVPRTGNM

Stu_RIOK2 FPQMISRDHKLANYERDADGIVNFFSRYFDIPSDDLP-SSLDSIQRTDDV

Sro_RIOK2 FPQMISRDHKLANYERDAEGVVNFFSRYFDIPLDDLP-SSLDSIKRIDDV

Sma_RIOK2 FPQMISRDHKLANYERDAEGVVNFFSRYFDIPLDDLP-SSLDSIKRIDDV

Sha_RIOK2 FPQMISRDHKLANYERDAEGVVNFFSRYFDIPLNDLP-SSLDSIKRVGYV

Smat_RIOK2 FPQMISRDHKLADYERDAEGVVNFFSRYFDIPLNDLP-SSLDSIKRVDYV

Scu_RIOK2 FPQMISRDHKLANYERDAEGVVNFFSRYFDIPLNDLP-SSLDSIKRVDYV

Sint_RIOK2 FPQMISRDHKLANYERDAEGVVNFFSRYFDIPLNDLP-SSLDSIKRVDYV

Smar_RIOK2 FPQMISRDHKLANYERDAEGVVNFFSRYFDIPLNDLP-SSLDSIKRVDYV

Fma-RIOK-2 FPQMISRDHETAEYERDGNALTSYFGRFFTIEETDLP-QLLSEVERVADV

Fhe-RIOK-2 FPQVISRDHETAEYERDGNALTSYFGRFFTIEELDLP-QLLSEVERVADV

Fgi-RIOK-2 FPQVISRDHETAEYERDGNALTSYFGRFFTIEELDLP-QLLSEVERVADV

Ovi_RIOK2 FPQMISRAHPTAEYRRDAEGIVSFFSRFFEIPEEDLP-LSLSEIKRTAYL

Csi_RIOK2 FPQMISRAHPTAEYRRDAEGIVSFFSRFFEIPEEDLP-LSLSEITRTAYL

Cgi_RIOK2 DVEVSASGFAAEEFNIRGEEDEDNDSVENEKQRRLIKEKVKRQMKKKAAV

Hna_RIOK2 DIKLKAPGYSVQKKSKDTKQVDDSEFLVGTVAEENAKSLARENASISTQR

Hdi_RIOK2 DIELKAPGYPVQKKSKDTKQADDSELLVGTVAQEEIRERKKREDRRRAQH

Hmic_RIOK-2 DIKLKAPGYSVQKKSKDIKQVDDSELLVGTVAQEEIRERKRREDRRRAQR

Mco_RIOK2 DVDLKAPGYIREKPSKRSAVVEDSDLLVGTVARDEVRERRKREEKKRMQN

Tta_RIOK2 DVELKAPGYPNQRSQKRNKSMDDSELLVGTVAREEVRERKKREERKRLQN

Tsol_RIOK-2 DVELRAPGYPNQKPPKRTKRMDDSELLVGTVAREEVRERKKREERKRTQN

Tas_RIOK2 DVELRAPGYPNQKPQKRTKRMDDSELLVGTVAREEVRERKKREERKRTQN

Egra_RIOK-2 DVELKAPGYPNQKPQKKTERIDDSELLVGTVAREEVRERRKREGRKRIQN

Emul_RIOK-2 DVELKAPGYPNQKPQKKTERIDDSELLVGTVAREEVRERRKREGRKRIQN

Stu_RIOK2 DVHLKAPGYISKNATNRHSRHTVEDSLLGSVAREEIRERNRRERRHQQQV

Sro_RIOK2 DLHLKAPGYISKNATNRHSQHTTEDSLLGPVAREEIRERNRRERRHQKQV

Sma_RIOK2 DLHLKAPGYISKNATNRHSQHTTEDSLLGPVAREEIRERNRRERRRQKQV

Sha_RIOK2 DVHLKAPGYISKNATNRHSKHTIEDSLLGSVAREEIRERNRRERRHQKQV

Smat_RIOK2 DVHLKAPGYISKNATNRHSKHTIEDSLLGSVAREEIRERNRRERRHQKQV

Scu_RIOK2 DVHLKVPGYISKNATNRHSKHTIEDSLLGSVAREEIRERNRRERRHQKQV

Sint_RIOK2 DVHLKAPGYISKNATNRHSKHTIEDSLLGSVAREEIRERNRRERRHQKQV

Smar_RIOK2 DVNLKAPGYISKNATNRHSKHTIEDSLLGPVAREEIRERNRRERRHQKQV

Fma-RIOK-2 DVQVKAPGYQSKHTSRHEKRPTVREILLGATSRREIRAKHRCEQRQREQI

Fhe-RIOK-2 DVQVKAPGCQSKHISRHEKRPTVREILLGTTSHREIRVKHRREQRQQEQI

Fgi-RIOK-2 DVQVKAPGCQSKHISRHEKRPTVREILLGTTCHREIRAKYRREQRQQEQI

Ovi_RIOK2 DVEVRAPGFPSKKFQRRGRTRDDHQVLLGATSREEIRARHRREKRQQEQM

Csi_RIOK2 DVEVKAPGFPSKKFQRRGRTRDDHQVLLGATSGRKARARAREHVRTKHVK

Cgi_RIOK2 QESRRIRK

Hna_RIOK2 EFLLAVKR

Hdi_RIOK2 EFLLGVKR

Hmic_RIOK-2 EFLLAVKR

Mco_RIOK2 EFRLRIKR

Tta_RIOK2 EFRLRIKR

Tsol_RIOK-2 EFRLRIKR

Tas_RIOK2 EFRLRIKR

Egra_RIOK-2 EFRLRIKR

Emul_RIOK-2 EFRLRIKR

Stu_RIOK2 DFNRQIKR

Sro_RIOK2 DFNRQIKR

Sma_RIOK2 DFNRQIKR

Sha_RIOK2 DFNRQIKR

Smat_RIOK2 DFNRQIKR

Scu_RIOK2 DFNRQIKR

Sint_RIOK2 DFNRQIKR

Smar_RIOK2 DFNRQIKR

Fma-RIOK-2 RLQQRIKR

Fhe-RIOK-2 RLQQRIKR

Fgi-RIOK-2 KLQQRIKR

Ovi_RIOK2 NFQRNVKR

Csi_RIOK2 TEHERLKK

;

end;

begin mrbayes;

log start replace filename = mrbayes.log;

prset aamodelpr=mixed;

lset rates=invgamma;

prset ratepr=variable;

showmodel;

mcmc ngen=2000000 printfreq=10000 samplefreq=100

nchains=4 diagnfreq=1000

nruns=2 nperts=2;

sumt relburnin=yes burninfrac=0.25 contype=halfcompat;

sump relburnin=yes burninfrac=0.25;

log stop;

end;

**Supplementary Data 4**: Representative metazoans included in this study, and the accession numbers of their genome sequences.

| Taxonomic group | Species | GenBank Assembly or  Bioproject IDs |
| --- | --- | --- |
| Cnidaria | *Nematostella vectensis* | GCA_000209225.1 |
|  | *Hydra vulgaris* | GCA_000004095.1 |
|  | *Acropora digitifera* | GCA_000222465.1 |
|  | *Alatina moseri* | GCA_000260875.1 |
| Porifera | *Amphimedon queenslandica* | GCA_000090795.1 |
| Crustacea | *Daphnia pulex* | GCA_000187875.1 |
|  | *Eurytemora affinis* | GCA_000591075.1 |
| Insecta | *Drosophila melanogaster* | GCA_000001215.2 |
|  | *Bombyx mori* | GCA_000151625.1 |
|  | *Tribolium castaneum* | GCA_000002335.2 |
| Mollusca | *Lottia gigantea* | GCA_000327385.1 |
|  | *Biomphalaria glabrata* | GCA_000457365.1 |
|  | *Aplysia californica* | GCA_000002075.2 |
|  | *Crassostrea gigas* | GCA_000297895.1 |
|  | *Mytilus galloprovincialis* | GCA_000715055.1 |
| Annelida | *Helobdella robusta* | GCA_000326865.1 |
|  | *Capitella teleta* | GCA_000328365.1 |
| Chordata | *Homo sapiens* | GCA_000001405.15 |
|  | *Gallus gallus* | GCA_000002315.2 |
|  | *Branchiostoma floridae* | GCA_000003815.1 |
|  | *Strongylocentrotus purpuratus* | GCA_000002235.2 |
|  | *Saccoglossus kowalevskii* | GCA_000003605.1 |
| Platyhelminthes | *Clonorchis sinensis* | GCA_000236345.1 |
|  | *Opisthorchis viverrini* | GCA_000715545.1 |
|  | *Schistosoma haematobium* | GCA_000699445.1 |
|  | *Schistosoma Japonicum* | GCA_000151775.1 |
|  | Schistosoma mansoni | GCA_000237925.2 |
|  | *Echinococcus multilocularis* | GCA_000469785.1 |
|  | *Echinococcus granulosus* | GCA_000524195.1 |
|  | *Taenia solium* | PRJNA183343 |
|  | *Hymenolepis microstoma* | GCA_000469805.1 |
|  | *Schmidtea mediterranea* | GCA_000691995.1 |
| Nematoda | *Caenorhabditis elegans* | GCA_000002985.3 |
|  | *Brugia malayi* | GCA_000002995.2 |
|  | *Caenorhabditis brenneri* | GCA_000143925.2 |
|  | *Trichinella spiralis* | GCA_000181795.2 |
|  | *Ascaris suum* | GCA_000298755.1 |
|  | *Panagrellus redivivus* | GCA_000341325.1 |
|  | *Haemonchus contortus* | GCA_000469685.1 |
|  | *Trichuris trichiura* | GCA_000172435.1 |
|  | *Meloidogyne hapla* | GCA_000180415.1 |
|  | *Pristionchus pacificus* | GCA_000180635.1 |
|  | *Loa loa* | GCA_000183805.1 |
|  | *Strongyloides ratti* | GCA_000208845.1 |

**Supplementary Data 5**: SSU cytoplasmic associating proteins presence/absence in human, C.elegans and S. haematobium species genomes.

| ***H.sapiens*** | ***C. elegans*** | ***S.haematobium*** |
| --- | --- | --- |
| LTV-1 | T23D8.3 | MS3_01198 |
| ENP-1/Bystin | byn-1 | A_06655 |
| NOB-1 | nob-1 | A_00775 |
| TSR-1 | tsr-1 | A_06601 |
| DIM-1 | dim-1 | A_02243 |
| PNO-1 | Y53C12B.2 | A_04722 |

**Supplementary Data 6**. Ligand-interacting residues of the nucleotide binding pocket determined for the three-dimensional structures of RIOK-1 and RIOK-2 of *Homo sapiens,* *Schistosoma haematobium* and *Taenia solium*. In case of RIOK-1 from *H. sapiens*, the available crystal structure of *Hsap*-RIOK-1:ADP (PDB accession code 4otp) was analysed. For all other proteins, models were generated as described in the main text.

|  | *Homo sapiens* | *Schistosoma haematobium* | *Taenia solium* |
| --- | --- | --- | --- |
| **RIOK-1** | K208, S278, I280, N329, F341 | K98, E99, I102, F188, P195, A196, P197, Y243, N244, I255, D256 | S85, V92, M175, T176, I178, N234, Q249 |
| **RIOK-2** | G104, K105, I109, A121, K123, I191, P195, N233, I245 | E87, V90, V121, K123, S189, Y190, F240, N241, I263 | E118, L123, P216, M228, S229, L231, F280, N281, I314, D315 |

**Supplementary Data 7.** Amino acid substitutions in *Schistosoma haemaotobium* and *Taenia solium* nucleotide binding sites of RIOKs as compared to the *Homo sapiens* protein. The residue numbers given refer to the *H. sapiens* protein.

|  | *Schistosoma haematobium* | *Taenia solium* |
| --- | --- | --- |
| **RIOK-1** | I194I  L289P | L289K |
| **RIOK-2** | I109V  I111V  I235M | I109V  I111L  A121V  I191L  I235L |
